# From Amazon to Hospital Nurse

**Published:** 1918-11-30

**Authors:** 


### WOMEN OF ACTION IN PAST WARS.

### From Amazon to Hospital Nurse.

We hear so much of women's work to-day that we are tempted to imagine that—at least before Florence Nightingale—their part in war and action was to sit at home, and wait. As a matter of fact the most casual lifting of the veil, even for a moment, reveals an interesting list of heroines who have fought, worked, and healed in ancient wars.

First come the Amazons, true warriors, scorning pain and death, and credited with burning off their right breasts in order to draw their bows freely. These must not be dismissed as mythical creatures, for at Belmonte Professor d'Allosso recently discovered the tombs of two warrior women of the Iron Age, whence he infers that "the existence of Amazon heroines is not a poetic invention, but a historic reality." It was the Amazons who gave their name to the greatest of rivers, on whose banks the Spanish explorer, Orellana, met from 1540-41 with stubborn resistance from native women. The women of Carthage at the great siege of 146 B.C. cut off their hair to make catapults, and when the Romans entered the city fought desperately in the streets for six days and nights. Tacitus tells how the British warrior queen, Boadicea, after her husband was killed, rallied her tribesmen, the Iceni, around her, took London and St. Albans from the Romans, and slew 70,000 legionaries before she was finally conquered *circa* 60 A.D. Celtic women habitually fought in battle until the sixth century, when St. Columba, horrified by the atrocities which he had seen in Ireland, had the practice condemned at the Synod of Drumceatt. Nevertheless, many women have since taken up arms in times of peril. In 1338 Black Agnes held Dunbar Castle for nineteen weeks, till the English were forced to raise the siege. In the following century Joan of Arc, an inspired peasant girl, drove the invader from France. During the Civil War King Charles I. was aided by many loyal women, some of whom held their castles for him, and that keen observer, Butler, tells in "Hudibras" how

> "From ladies down to oyster wenches
> Laboured like pioneers in trenches."

In the nineteenth century Garibaldi's wife, Anita, a Brazilian, by the way, followed him, like another Lady Burton, through his desperate ventures till the very hour of her death in 1849, and incessantly tended and encouraged her husband and his devoted followers.

Among women workers we must not forget the mothers of Sparta and the Roman matrons, the end of whose existence was to fit their sons for war. And no Englishwoman can forget Deborah, the prophetess of Israel, whose stirring hymn of triumph the Authorised Version has re-created in our own language. In striking contrast comes Geneviève, the patron saint of Paris, who assured the terror-stricken citizens that Attila and his Huns would not come upon them, and later, during the Frankish invasion under Childeric, journeyed from town to town and returned to starving Paris with twelve shiploads of provisions. Are modern girls no longer taught the tale of the Countess of Buchan, who, in the absence of her brother, the Earl of Fife, crowned King Robert the Bruce at Scone with a slender golden circlet from the head of a saint's statue? A price was set on her head, and after many wanderings she was seized and imprisoned by the English at Berwick, where at length she died. Then there is the tale of the mother of King René of Anjou, Yolande d'Aragona, who ruled Anjou and Provence while her husband fought for his rights as King of Naples; Yolande championed the cause of Jeanne d'Arc from the beginning, and herself clothed "la Pucelle" in armour. René's daughter, Margaret of Anjou, the unlucky wife of Henry VI., showed great military gifts and administrative ability during the Wars of the Roses. And we cannot omit Queen Elizabeth, Queen Christina of Sweden, and the Empress Catherine of Russia, who were such women of action that their sex has been questioned before now—in an attempt to explain their masculine qualities.

If we would see something of the feminine healers of the past, it seems that not till towards the end of the Thirty Years' War, which lasted from 1618 to 1648, did a prominent saint and hero arise who saw how valuable the work of women might be in alleviating public distress. St. Vincent de Paul, appalled by the misery of his country, called all classes of the women of France to his aid. His Sisters of Charity sheltered hundreds of women and girls from the war zone; they officered parties to bury the dead and clean away the filth by the roadsides. During the Fronde they distributed the saint's "potages économiques" daily to 1,300 "bashful poor." Anne of Austria employed them to nurse the wounded in her hospital at Fontainebleau. Then the Queen of Poland asked St. Vincent to send three Sisters of Charity to nurse the wounded in her country, and theirs was the honour to be the first women to tend the wounded actually on the field of battle. The Order spread over the world; its "Angels of the Battlefield" succoured the Federals and Confederates alike during the American Civil War, and their call for help from the Crimea prompted Florence Nightingale to set out on her first mission.

